# *Wolbachia 16S* rRNA haplotypes detected in wild *Anopheles stephensi* in eastern Ethiopia

**DOI:** 10.1186/s13071-022-05293-9

**Published:** 2022-05-24

**Authors:** Elizabeth Waymire, Sowmya Duddu, Solomon Yared, Dejene Getachew, Dereje Dengela, Sarah R. Bordenstein, Meshesha Balkew, Sarah Zohdy, Seth R. Irish, Tamar E. Carter

**Affiliations:** 1 Department of Biology, Baylor University, Waco, TX USA; 2 Jigjiga University, Jigjiga, Ethiopia; 3 Dire Dawa University, Dire Dawa, Ethiopia; 4 PMI VectorLink Ethiopia Project, Abt Associates, Addis Ababa, Ethiopia; 5 Department of Biological Sciences, Vanderbilt University, Nashville, TN USA; 6 U.S. President’s Malaria Initiative and Entomology Branch, Centers for Disease Control and Prevention, Atlanta, GA USA

**Keywords:** *Anopheles stephensi*, *Wolbachia*, Horn of Africa, Malaria, Vector control, Disease control

## Abstract

**Background:**

About two out of three Ethiopians are at risk of malaria, a disease caused by the parasites *Plasmodium falciparum* and *Plasmodium vivax. Anopheles stephensi*, an invasive vector typically found in South Asia and the Middle East, was recently found to be distributed across eastern and central Ethiopia and is capable of transmitting both *P. falciparum* and *P. vivax*. The detection of this vector in the Horn of Africa (HOA) coupled with widespread insecticide resistance requires that new methods of vector control be investigated in order to control the spread of malaria. *Wolbachia*, a naturally occurring endosymbiotic bacterium of mosquitoes, has been identified as a potential vector control tool that can be explored for the control of malaria transmission. *Wolbachia* could be used to control the mosquito population through suppression or potentially decrease malaria transmission through population replacement. However, the presence of *Wolbachia* in wild *An. stephensi* in eastern Ethiopia is unknown. This study aimed to identify the presence and diversity of *Wolbachia* in *An. stephensi* across eastern Ethiopia.

**Methods:**

DNA was extracted from *An. stephensi* collected from eastern Ethiopia in 2018 and screened for *Wolbachia* using a *16S* targeted PCR assay, as well as multilocus strain typing (MLST) PCR assays*.* Haplotype and phylogenetic analysis of the sequenced *16S* amplicons were conducted to compare with *Wolbachia* from countries across Africa and Asia.

**Results:**

Twenty out of the 184 mosquitoes screened were positive for *Wolbachia,* with multiple haplotypes detected. In addition, phylogenetic analysis revealed two superclades, representing *Wolbachia* supergroups A and B (bootstrap values of 81 and 72, respectively) with no significant grouping of geographic location or species. A subclade with a bootstrap value of 89 separates the Ethiopian haplotype 2 from other sequences in that superclade.

**Conclusions:**

These findings provide the first evidence of natural *Wolbachia* populations in wild *An. stephensi* in the HOA. They also identify the need for further research to confirm the endosymbiotic relationship between *Wolbachia* and *An. stephensi* and to investigate its utility for malaria control in the HOA.

**Graphical Abstract:**

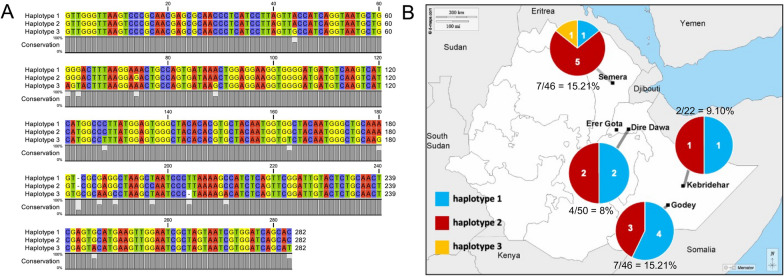

**Supplementary Information:**

The online version contains supplementary material available at 10.1186/s13071-022-05293-9.

## Background

Over 229 million cases of malaria, primarily caused by *Plasmodium falciparum* and *Plasmodium vivax*, were reported worldwide in 2019 [1]. Although the prevalence of malaria in Ethiopia is not as high as in other countries in sub-Saharan Africa, the threat of increased malaria transmission is present as a new malaria vector has invaded eastern Ethiopia. *Anopheles stephensi*, a typical South Asian or Middle Eastern vector, was first detected in Djibouti in the Horn of Africa (HOA) in 2013 [2]. Since then, it has been detected in southeastern Ethiopia in 2016 [3], Sudan in 2016 [4], and Somalia in 2019 [4]. In 2018, *An. stephensi* was confirmed to be distributed across eastern Ethiopia [5]. It has been demonstrated that *An. stephensi* is capable of transmitting both *P. falciparum* and *P. vivax* in eastern Ethiopia and the establishment of this vector could increase the prevalence of *P. falciparum* cases by 50% in areas found to be suitable if no additional intervention occurs [6, 7]. With the knowledge that *An. stephensi* can transmit both *Plasmodium* species in eastern Ethiopia, strategies for the control or elimination of *An. stephensi* in the HOA are being investigated. Insecticide resistance has already been identified in *An. stephensi* in eastern Ethiopia [8, 9], requiring alternative methods of vector control such as the use of *Wolbachia pipientis*.

*Wolbachia pipientis,* more commonly referenced as *Wolbachia,* is an endosymbiotic bacterium of invertebrates that is classified into different supergroups based on major phylogenetic diversions. All *Wolbachia* in mosquitoes belong to supergroups A and B [10]. *Wolbachia* has been identified as a resource to address vector-borne diseases in *Aedes* mosquitoes through two main methods: population suppression [11] and population replacement [12, 13]. Both methods are based on the mechanism of cytoplasmic incompatibility (CI). When an uninfected female mosquito mates with a male mosquito infected with *Wolbachia*, embryonic death occurs, and the female’s larvae are inviable. In population suppression, male mosquitoes infected with *Wolbachia* are released on a large scale to decrease the population size, thus decreasing the transmission of vector-borne diseases. Population replacement involves releasing both male and female mosquitoes with *Wolbachia* infection into a population of *Wolbachia*-free, disease-carrying mosquitoes [14].

Generally, the relationship between *Wolbachia* infection and *Plasmodium* infection in field *Anopheles* species is not well studied. Kambris et al. [15] and Hughes et al. [16] have shown that in the lab, *Anopheles gambiae* can be infected with wMelPop and wAlbB, and that *Plasmodium berghei* and *P. falciparum* development is inhibited with both *Wolbachia* strains. In field populations of *Anopheles coluzzii* and *An. gambiae*, Shaw et al. [17] and Gomes et al. [18] demonstrated that *Wolbachia* infection correlated negatively with *Plasmodium* development. This is indicative of potential for *Wolbachia*-based interventions, but more research is needed.

In *An. stephensi* specifically, *Wolbachia* infections in lab populations have been briefly studied. Bian et al. [19] were able to stably infect *An. stephensi* with wAlbB. They showed that maternal transmission and CI induction were successful, and observed resistance to *P. falciparum* infection in the lab strain of *An. stephensi*. Chen et al. [20] showed that *An. stephensi* can be stably infected by wAlbB and that there was no change in the microbiome of the mosquitoes upon *Wolbachia* infection. These results indicate that lab strains of *An. stephensi* can hold stable infections of *Wolbachia*, which may mean that field populations are susceptible to infection, although further research is needed [18].

The limited study of *Wolbachia* infection in *An. stephensi* has mainly been in lab populations. We need to determine whether *Wolbachia* infection is present in order to begin evaluating the potential of *Wolbachia*-based control strategies for malaria transmission in *An. stephensi* in eastern Ethiopia. Furthermore, the strain of *Wolbachia* in the wild host population needs to be determined, to establish which population control strategy would function best, should a validated *Wolbachia*-based malaria control tool become achievable [13]. *Wolbachia* is typically detected via DNA or RNA extraction from the host invertebrate and targeting multiple genes to determine infection. These genes include the *16S*-encoding gene, the surface protein-encoding gene (Wolbachia surface protein [*wsp*]), and the five multilocus strain typing (MLST) genes (*ftsZ*, *fbpA*, *hcpA*, *coxA*, and *gatB*) [10]. Previous studies have depended solely on *Wolbachia 16S* for confirming infection [18, 19]; however, more recent research has indicated that multiple genes must be detected in order to confirm infection [20]. Very few *Anopheles* species have been screened for *Wolbachia*, with proven infection occurring only in two highly diverged species in sub-Saharan Africa [21]. There has also been significant difficulty in many studies with amplifying the genes in MLST. Gomes et al. were only able to amplify three out of five genes, and Jeffries et al. were unable to amplify any MLST genes in multiple sample groups [18, 20]. This may indicate that environmental contamination is present or that divergence among these genes has emerged, precluding the use of the standard MLST primer set [18, 22, 23].

Since the detection of *An. stephensi* in the HOA, no investigation regarding the possibility of *Wolbachia* endosymbiosis has been conducted in this population. One study surveyed *An. stephensi* for *Wolbachia* in Tamil Nadu, India, and reported the detection of *16S Wolbachia* DNA in a portion of their mosquitoes [24]. Still, more information is needed to confirm whether *Wolbachia* is a true endosymbiont of *An. stephensi*, as *Wolbachia* in *An. stephensi* in the Middle East and the rest of South Asia has not been investigated.

In order to evaluate *Wolbachia* as a potential control method for malaria transmission, we first need to know whether *Wolbachia* is present in *An. stephensi* in eastern Ethiopia. In addition, the detection of *Wolbachia* DNA could provide a basis for using it in the complementary phylogeographic analysis of the *An. stephensi* invasion. In this study, we used *Wolbachia 16S* ribosomal RNA (rRNA)-, *wsp*-, and MLST-targeted polymerase chain reaction (PCR) assays to detect *Wolbachia* in *An. stephensi* from eastern Ethiopia.

## Methods

### Sampling of *An. stephensi*

*Anopheles stephensi* surveys were conducted from August through November 2018 in Semera, Godey, Kebridehar, Erer-Gota, and Dire Dawa. Details about the collection sites and approach have been described previously [5]. Briefly, adult mosquitoes were collected via pyrethrum spray catch (PSC) and Centers for Disease Control and Prevention (CDC) light traps. The collected mosquitoes were sorted between culicines and *Anopheles*, and the latter were further sorted to distinguish *An. stephensi* from other *Anopheles* species using a standard morphological key [25] and a key prepared by Coetzee (subsequently published in 2020 [26]). Analysis of *Plasmodium* detection was conducted previously, and no wild-caught adult mosquitoes contained *Plasmodium falciparum* or *P. vivax* DNA [5].

Larvae and pupae of *Anopheles* were also collected from larval habitats including artificial water storage containers, freshwater pools, discarded tires, and plastic containers. Larvae were reared in field insectaries using water taken from larval habitats, feeding them with baker’s yeast and exposing them to sunlight during the day. Pupae were transferred into adult emergence cages and adults were morphologically identified using the same keys [25, 26]. The numbers of wild-caught adults and reared larvae are documented in Additional file 1: Table S1.

### DNA extraction and molecular identification of mosquitoes

DNA was extracted from either the abdomens or whole bodies of *An. stephensi* mosquitoes, selected at random, using the DNeasy Blood & Tissue Kit (Qiagen, Valencia, CA, USA). PCR was performed for each individual mosquito, targeting the nuclear internal transcribed spacer 2 (ITS2) region and the mitochondrial cytochrome *c* oxidase subunit 1 (*COI*). The reagent components and final concentrations for the PCR assays were 1× Promega Hot Start Master Mix (Promega, Madison, WI, USA) and 0.5 mM for both primers, plus 1 µl of isolated DNA template. A region including the ITS2 gene was amplified via PCR using universal primers as described previously [27]. Updated annotations show that identification via these primers is based on a portion of the sequence that includes 28S in the ITS2 assay [28]. The PCR temperature protocol consisted of 95 °C for 1 min, 30 cycles of 95 °C for 30 s, 48 °C for 30 s, and 72 °C for 1 min; followed by 72 °C for 10 min. All samples successfully amplified ITS2. A region including the *COI* gene was amplified via PCR using universal primers as described previously [3]. The PCR temperature protocol consisted of 95 °C for 1 min, 30 cycles of 95 °C for 30 s, 48 °C for 30 s, and 72 °C for 1 min; followed by 72 °C for 10 min.

### Molecular *Wolbachia* detection

The *Wolbachia 16S* rRNA-encoding gene (*16S*)*,* and the *Wolbachia* surface protein encoding gene (*wsp*) were amplified to detect *Wolbachia* in the mosquitoes. A positive control of extracted genomic DNA from *Wolbachia*-infected *Drosophila melanogaster* (provided by The Wolbachia Project at Vanderbilt University) was used to troubleshoot PCR protocols and ensure the PCR successfully amplified *Wolbachia 16S, wsp,* and the genes within MLST. A negative control of no genomic DNA (gDNA) template was used to ensure no contamination of the PCR reagents. The reagent components and final concentrations for the PCR assays were 1X Promega Hot Start Master Mix (Promega, Madison, WI, USA) and 0.4 mM for each primer *16S* and *wsp*, plus 4 µl and 2 µl of isolated DNA template for *Wolbachia 16S*, and *wsp*, respectively. For the *16S* assay, a nested protocol was used.

The first set of *Wolbachia 16S* primers, as described in Werren and Windsor, amplified an un-nested 438-base-pair (bp) fragment [29]. The PCR cycling protocol was as follows: 95 °C for 2 min, 2 cycles of 95 °C for 1 min, 60 °C for 1 min, and 72 °C for 1 min, followed by 35 cycles of 95 °C for 30 s, 60 °C for 1 min, and 72 °C for 45 s, and lastly 72 °C for 5 min. For the nested reaction, specific internal primers were used as described by Shaw et al., which amplified a 412-bp fragment [17]. Two microliters of PCR product from the un-nested reaction was used in this reaction as template DNA. The PCR temperature protocol was as follows: 95 °C for 15 min, 35 cycles of 95 °C for 15 s, 66 °C for 25 s, and 72 °C for 30 s, followed by 72 °C for 5 min.

For the *wsp* assay, a 546 bp region including the gene was amplified using primers as described in Zhou et al. [30]. The PCR temperature protocol was as follows: 94 °C for 2 min, then 35 cycles of 94 °C for 1 min, 55 °C for 1 min, and 72 °C for 1 min; followed by 72 °C for 5 min. All primers used are listed in Additional file 1: Table S2. All PCR products were run on a 2% agarose gel, and the gel was visualized using a Gel Doc EZ imager (Bio-Rad Laboratories, Inc., Hercules, CA, USA) prior to Sanger sequencing by a commercial laboratory (Eurofins Genomics LLC).

### Multilocus strain typing

In addition to amplifying the *16S* and *wsp* genes, MLST was performed on each *16S*-positive sample to determine whether the *Wolbachia* infection was present. Five loci, including *ftsZ*, *fbpA*, *hcpA*, *coxA*, and *gatB*, were tested, using region-specific primers. The *ftsZ* universal assay amplified a 775 bp region [31], the *fbpA* assay amplified 429 bp, the *hcpA* assay amplified a 444 bp region, the *coxA* assay amplified a 402 bp region, and the *gatB* assay amplified a 369 bp region [10]. The final concentrations for the PCR assays were 1X Promega Hot Start Master Mix (Promega, Madison, WI, USA), 0.4 mM for each primer, plus 2 µl of isolated DNA template for each reaction.

The PCR cycling protocol for *coxA*, *hcpA*, and *gatB* consisted of 94 °C for 2 min, 36 cycles of 94 °C for 30 s, 54 °C for 45 s, and 72 °C for 1 min and 30 s; followed by 72 °C for 10 min. The PCR cycling protocol for *fbpA* consisted of 94 °C for 2 min, 36 cycles of 94 °C for 30 s, 59 °C for 45 s, and 72 °C for 1 min and 30 s; followed by 72 °C for 10 min. Lastly, the PCR cycling protocol for *ftsZ* universal consisted of 95 °C for 2 min, 35 cycles of 94 °C for 30 s, 58.2 °C for 1 min, and 72 °C for 1 min; followed by 72 °C for 5 min.

A nested protocol was used for all five MLST genes. The *hcpA*, *fbpA*, and *gatB* nested assays were used as previously described on the PubMLST website [32]. Two microliters of PCR product from the un-nested reaction was used in this reaction as template DNA. Nested PCR cycling conditions were as follows: 95 °C for 15 min, 50 cycles of 95 °C for 15 s, 55 °C for 30 s, and 72 °C for 1 min; followed by 72 °C for 5 min. The *ftsZ* and *coxA* nested assays were used as described previously and amplified products 424 bp and 357 bp in length, respectively [33]. The nested PCR protocol for *ftsZ* and *coxA* consisted of 94 °C for 5 min, 36 cycles of 94 °C for 15 s, 55 °C for 15 s, and 72 °C for 30 s; followed by 72 °C for 10 min. All primers used are listed in Additional file 1: Table S2. All PCR products were run on a 2% agarose gel, and the gel was visualized using a Gel Doc EZ imager (Bio-Rad Laboratories, Inc., Hercules, CA, USA).

### Sequence and phylogenetic analysis

Sequences were cleaned and analyzed using CodonCode (CodonCode Corporation, Centerville, MA, USA). *Wolbachia 16S*, *Wolbachia coxA*, and *An. stephensi COI* sequences were submitted as queries to the National Center for Biotechnology Information’s (NCBI) Basic Local Alignment Search Tool (BLAST) against the nucleotide collection under standard parameters (100 max target sequences, expect threshold 0.05, word size 28, optimized for highly similar sequences, not specific to any organism). This information was used to confirm that the amplicons produced were the *Wolbachia 16S* gene, *Wolbachia coxA* gene, or *An. stephensi COI*. An alignment of observed haplotypes and the nucleotide conservation in the *An. stephensi Wolbachia 16S* sequences were visualized on CLC Sequence Viewer 7.6 (CLC Bio Qiagen, Aarhus, Denmark).

Phylogenetic analysis was performed with the representative *16S* sequences collected from this study, as well as peer-reviewed, published *Wolbachia 16S* sequences from NCBI GenBank in anopheline mosquitoes with an outgroup of *Rickettsia japonica* (Additional file 1: Table S3) [18, 19, 33–38]. Phylogenetic analysis was also performed with the *coxA* sequences collected from this study, as well as peer reviewed, published *Wolbachia coxA* sequences from NCBI GenBank in anopheline mosquitoes with an outgroup of the *Wolbachia* endosymbiont of *Dirofilaria immitis* (Additional file 1: Table S3) [18, 33, 36]. An additional phylogenetic analysis was performed with the *Wolbachia 16S* sequences from sub-Saharan Africa used in the first analysis, the three *16S* haplotypes collected in this study, and four *16S* sequences from a study in pre-print from Tamil Nadu, India (Additional file 1: Table S3) [18, 24, 33, 36]. Lastly, phylogenetic analysis was performed on the *COI* genes from the *An. stephensi* that were *Wolbachia 16S*-positive, with an outgroup of *Anopheles maculatus* (Additional file 1: Table S3). Representative sequences from each location were determined by aligning sequences and eliminating non-distinct haplotypes. Alignments were created with MAFFT version 7 [39] and trimmed to 283 bp (*16S*; Fig. 2), 323 bp (*16S*; Additional file 1: Fig. S1)*,* 357 bp (*coxA*; Fig. 3), and 321 bp (*COI*; Fig. 4) using Mesquite version 3.61 [40]. Phylogenetic associations between all sequences were estimated using a maximum likelihood approach with RAxML [41]. The GTRGAMMA option that utilizes the general time-reversible (GTR) model of nucleotide substitution with the gamma model of the rate of heterogeneity was used. A total of 1000 runs were completed using the maximum likelihood criteria with rapid bootstrap analysis. The RAxML output was viewed in FigTree [42] with a root at the outgroup and transformed branches, and a phylogenetic tree image was made.

## Results

### *Wolbachia* detected in four of five survey sites

A total of 184 mosquito samples were tested, with 20 samples testing positive for *Wolbachia 16S* DNA in the nested PCR (Table 1). Two of these 20 samples were from wild-caught adult mosquitoes, whereas 18 were from laboratory-reared adults. Out of the 20 samples, seven samples were also positive in the un-nested PCR reaction (Table 2). The highest prevalence was found in Godey and Semera. One sample tested positive for *Wolbachia coxA* in the nested PCR reaction. No positives were detected using the *wsp* gene or the four other MLST genes.

**Table 1:** Prevalence of Wolbachia *16S* sequences in all *An. stephensi* samples. Samples were considered positive if a band appeared from the nested PCR and a high-quality sequence was produced

Site	No.	*16S*-positive samples	Prevalence (%)
Erer-Gota	20	0	0.0
Dire Dawa	50	4	8.0
Godey	46	7	15.2
Kebridehar	22	2	9.1
Semera	46	7	15.2
Total	184	20	10.9

**Table 2:** *16S*-positive *An. stephensi* from each site, number of samples with a positive band in the un-nested PCR reaction, and number of samples with a positive band in the *16S* nested PCR reaction

Site	Number of *An. stephensi* samples with a band in un-nested *16S* PCR	Number of *An. stephensi* samples with a band in nested *16S* PCR
Erer-Gota	0	0
Dire Dawa	1	4
Godey	4	7
Kebridehar	0	2
Semera	2	7
Total	7	20

### Multiple *16S* haplotypes detected

**Fig. 1:**
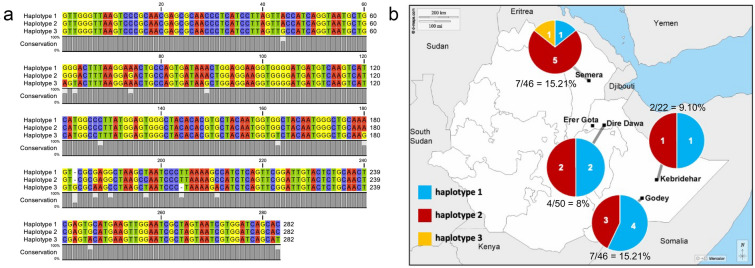
**a** Alignment of the three haplotypes observed from the *Wolbachia 16S* gene in eastern Ethiopia. Haplotypes were categorized as 1, 2, and 3. Nucleotide conservation is indicated by a gray bar below the alignment. **b** Haplotype frequency per collection site

After performing sequence analysis, three separate haplotypes of *Wolbachia 16S* were identified, designated as 1, 2, or 3. The most predominant haplotype was 2 (*n* = 11), followed by 1 (*n* = 8) and 3 (*n* = 1). Haplotype 1 was observed in Dire Dawa, Godey, Kebridehar, and Semera, with the highest number detected in Godey. Haplotype 2 was observed in Dire Dawa, Godey, Kebridehar, and Semera, with the highest number in Semera. Haplotype 3 was only present in one sample from Semera (Fig. 1).

Haplotypes 1 and 2 are the most similar, with 3 being more differentiated (16 nucleotide differences). Haplotype 3 has the highest number of single nucleotide substitutions or deletions in comparison with haplotypes 1 and 2. Even though there is a significant difference between haplotype 3 and haplotypes 1 and 2, most (94.33%) of the *16S* gene is conserved (Fig. 1a).

### *Wolbachia 16S in An. stephensi* falls into multiple clades

**Fig. 2:**
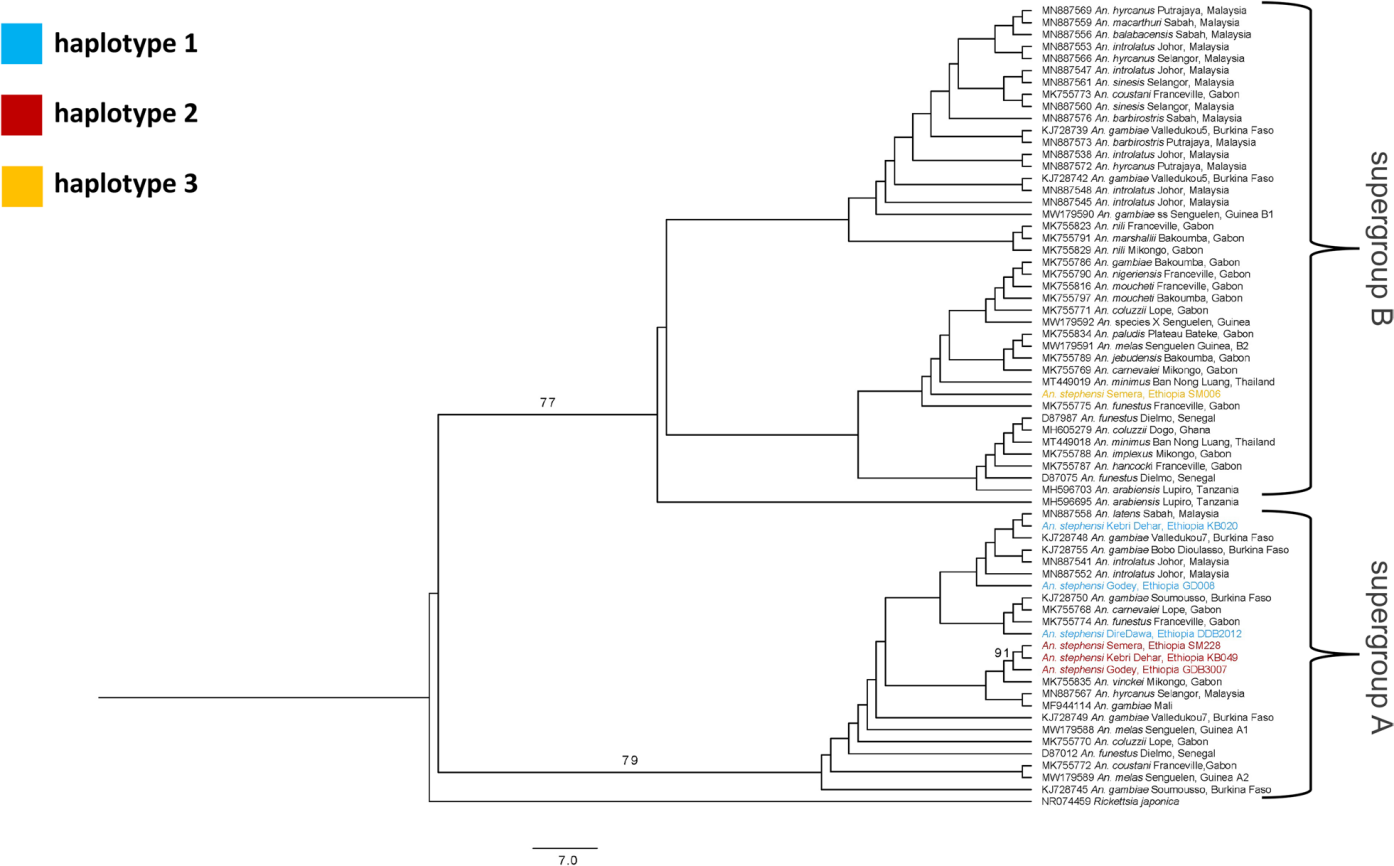
Phylogenetic tree of *Wolbachia 16S* in *Anopheles* species, with transformed branches. There were two major clades separated by significant bootstrap values 72 and 81. A subclade including haplotype 2 sequences from eastern Ethiopia was found to have significant support to be separate from the rest of the superclade. No geographic or species-specific clustering was found. The outlier sequence MH596695 did not follow the clustering of *Wolbachia* supergroup A or B, so it was not included in the distinction between these supergroups. *Rickettsia japonica* was used as the outgroup (NR074459). Haplotype 1 is designated by light blue font, haplotype 2 is designated by red font, and haplotype 3 is designated by orange font

Phylogenetic analysis revealed two superclades with bootstrap values of 81 and 72 representing *Wolbachia* supergroups A and B, respectively. Haplotype 3 only occurs in supergroup B, while haplotypes 1 and 2 occur in supergroup A. One of these superclades had a subclade composed of just haplotype 2 sequences from Ethiopia and received significant support separating this clade from the rest of the superclade (bs = 89). The *Wolbachia 16S* sequences from Ethiopia were in clades with sequences from both Africa and SE Asia and multiple *Anopheles* species. Overall, there was no clear clustering by geography or species (Fig. 2).

### *Wolbachia coxA *in eastern Ethiopia confirms the presence of *Wolbachia* in supergroup B

**Fig. 3:**
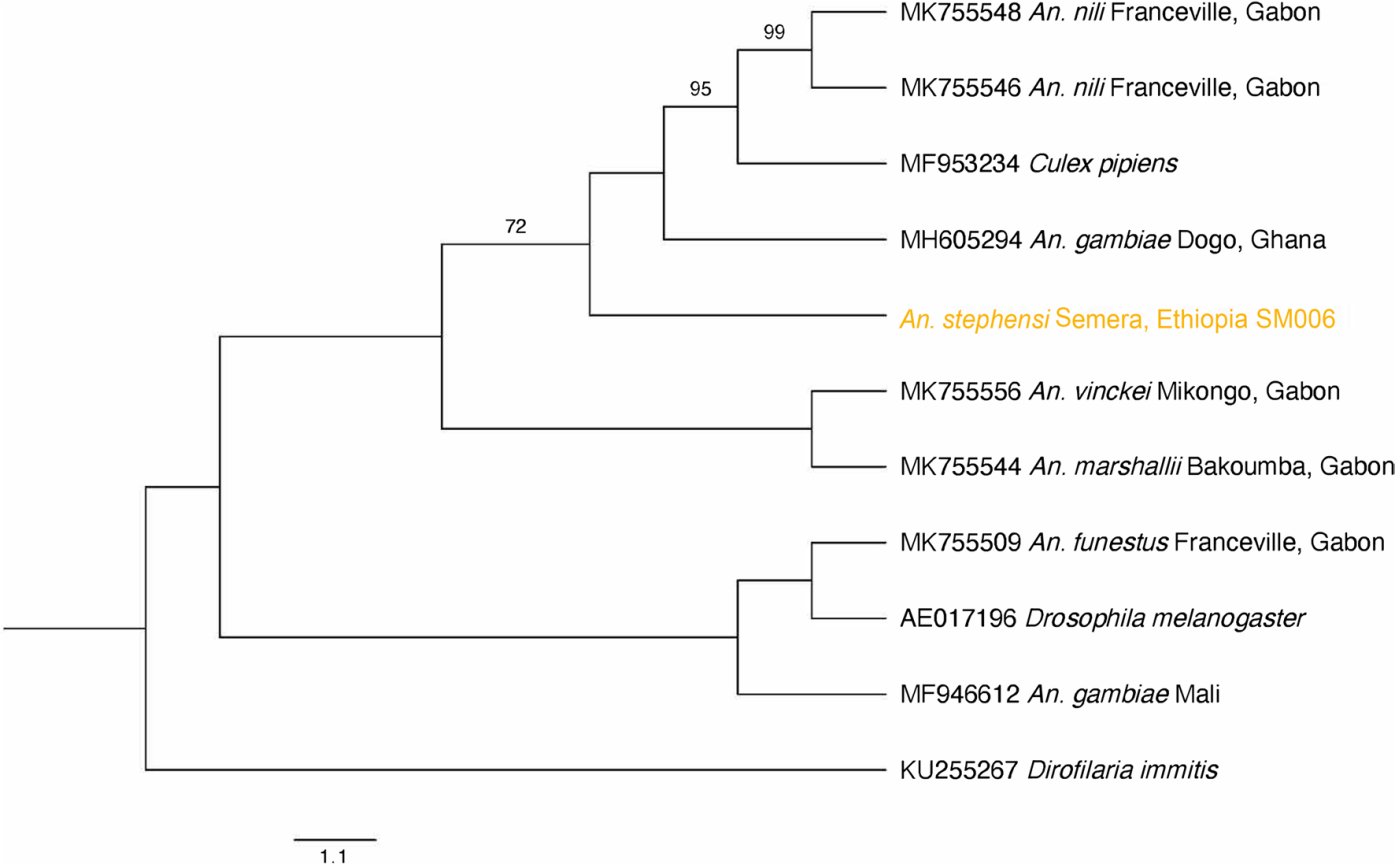
Phylogenetic tree of *Wolbachia coxA* in *Anopheles* species in sub-Saharan Africa, as well as *Wolbachia* strains from *Culex pipiens* and *Drosophila melanogaster* with transformed branches. We do not see separation into different supergroups; however, the sample from Ethiopia is significantly differentiated from the rest of the tree and clusters with samples known to be in supergroup B. Supergroup C *Wolbachia* from *Dirofilaria immitis* was used as the outgroup (KU255267). The sample from Ethiopia is designated in orange

Out of the five MLST genes, cytochrome *c* oxidase subunit 1 (*coxA*) was the only gene to be amplified, in the sample SM006. This sample is the only sample we found in supergroup B, and is the most distinguished haplotype, haplotype 3. We were able to amplify this gene three times from the same sample. Phylogenetic analysis showed that the sample from Ethiopia is significantly differentiated from the rest of the tree and clusters with samples known to be in supergroup B (Fig. 3).

### *Wolbachia *in *An. stephensi* from India falls into the same clade as haplotype 3 from eastern Ethiopia

To investigate whether *Wolbachia* in *An. stephensi* in eastern Ethiopia is similar to that found in the Middle East and South Asia, we performed a phylogenetic analysis of *Wolbachia 16S* sequences from Tamil Nadu, India, to those of eastern Ethiopia and sub-Saharan Africa. We again see separation into supergroups A and B. Supergroup B is represented in the top superclade and supergroup A is represented in the bottom superclade. The sample that is the strongest candidate for infection clusters with the sequences from India in supergroup B, but the sequences were not the same (Additional file 1: Fig. S1).

### *Wolbachia 16S *diversity and *An. stephensi COI* diversity

**Fig. 4:**
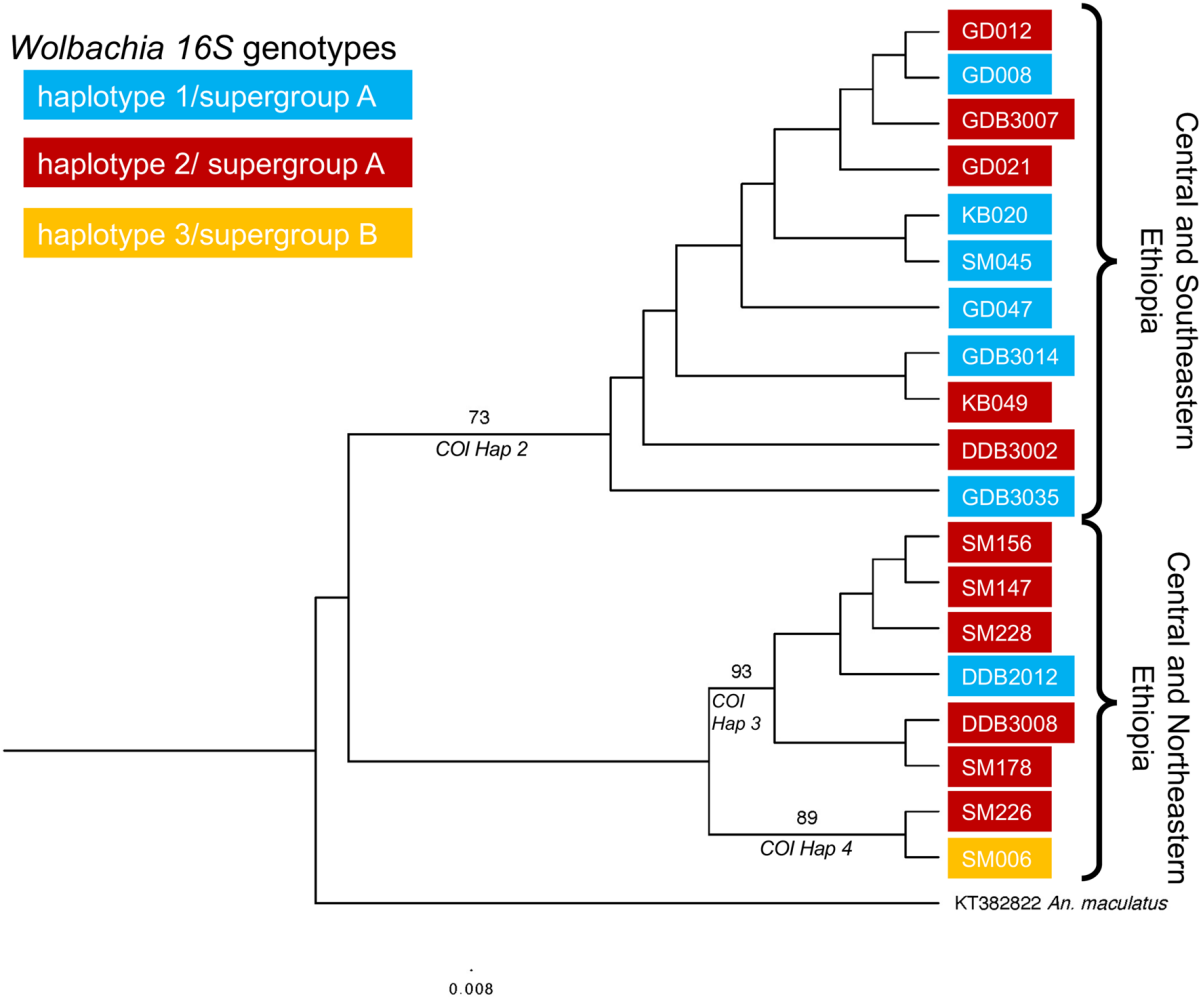
Phylogenetic analysis of *An. stephensi COI* in the *Wolbachia 16S*-positive *An. stephensi* with transformed branches. Each sample is colored by *Wolbachia* haplotype, where haplotype 1 is colored in blue, haplotype 2 is colored in red, and haplotype 3 is colored in yellow. Haplotypes 1 and 2 are representative of *Wolbachia* supergroup A, and haplotype 3 is representative of supergroup B. *Anopheles stephensi COI* haplotypes are designated on the clades that they represent according to Carter et al. [45]. *Anopheles maculatus* was used as an outgroup (KT382822). The sample sites are abbreviated as follows: Semera (SM), Dire Dawa (DD), Godey (GD), and Kebridehar (KB). Only 19 *An. stephensi* samples amplified *COI*, which was likely due to sequence divergence in the *COI* region in that mosquito

To investigate any correlation between *Wolbachia* diversity and *An. stephensi* diversity, we amplified the *COI* gene in all of our *Wolbachia 16S*-positive mosquitoes. The sample from Semera presents both the most *COI* diversity and the most *Wolbachia 16S* diversity. This is consistent with previous reports of mitochondrial diversity in the *An. stephensi* in eastern Ethiopia [5]. Samples from Semera (SM) occur in all three major clades in this tree. More southern sites, such as Godey (GD) and Kebridehar (KB), are in the topmost clade, and more northern and central sites such as Dire Dawa (DD) and Semera are mostly in the bottom two clades (Fig. 4). While we are unable to say that this pattern is definitive based on our sample size, we predict that if we were to obtain a larger sample set, we would see this trend separate our samples out more distinctively.

## Discussion

The data presented here confirm *Wolbachia 16S rRNA* molecular detection in *An. stephensi* and provide preliminary evidence of *Wolbachia* infection in this species. *Wolbachia 16S* DNA was detected in about 10% of samples across all collection sites. Three haplotypes were detected across all the sites with *Wolbachia 16S*-positive samples. Haplotypes 1 and 2 (supergroup A) were the most similar, with haplotype 3 (supergroup B) being the most dissimilar. Haplotype 2 was the most predominant haplotype, with 11 samples. Semera presented with the most diversity, exhibiting all three haplotypes. It should be noted that the number of positive *Wolbachia* samples was relatively low at each site, thus conclusions about the correlation between geographic location in Ethiopia and *Wolbachia 16S* diversity should be followed up with a larger sample set. Detection of *Wolbachia coxA* confirmed one of our samples, SM006, as a part of supergroup B. This provides an additional line of evidence of infection in the case of sample SM006, as most contamination scenarios can be eliminated.

Phylogenetic analysis revealed no significant clustering of *Wolbachia 16S rRNA* sequences with host *Anopheles* species or geographical regions. We predicted that there may be clustering of *Wolbachia 16S* based on geographic region or relatedness of the vector, but this was not observed. Phylogenetic analysis also revealed no significant relationship between *Wolbachia 16S* in India and that of eastern Ethiopia. Thus, there does not appear to be any specificity of *16S* haplotype to *Anopheles* species or geographic region. Lastly, phylogenetic analysis of *An. stephensi COI* colored by *Wolbachia 16S* haplotype revealed possible patterns of *Wolbachia* diversity. There is separation between southeastern Ethiopia and central/northeastern Ethiopia. Most of the sequences in *Wolbachia 16S* haplotype 1 appear in southeastern Ethiopia and most of the sequences in *Wolbachia 16S* haplotype 2 appear in central/northeastern Ethiopia. More samples would be needed in order to confirm any patterns observed.

The prevalence of detection found here is similar to that found in a study of the *An. gambiae* complex in Guinea, which was around 11% in *Anopheles melas* via MLST [20]. The authors of that study suggested that using a nested *16S* approach is problematic, citing a study investigating the presence of *Wolbachia 16S* in the *An. gambiae* genome. The authors discussed the establishment of the nested *16S* PCR protocol, and how it produces unreliable and non-replicable results [22]. We see in our study that only seven out of 20 total *16S*-positive samples showed a band in the un-nested PCR. All quality sequences were produced from nested PCR products, which raises the question of whether nested PCR should be used to determine *Wolbachia* infection.

The density of infection is currently unknown in *Anopheles* species. In a previous study, infections found in *An. demeilloni* and *An. moucheti* were described as high-density; however, corresponding data show *Wolbachia 16S* representing from less than 20% to just over 80% of the total microbiome. This indicates that densities in a species could vary, which would mean that the nested protocol may need to be used in the case of lower-density infections or co-infection with another bacterium such as *Asaia* [20]. As this method does not provide a clear measure of the number of bacteria, we suggest incorporating quantitative PCR (qPCR) and fluorescence in situ hybridization (FISH) in the future.

Questions about whether the detection of *Wolbachia* DNA in *An. stephensi* populations from this study is indicative of an actual infection can be further examined by comparing the diversity of the *Wolbachia* detected in the recently introduced population of *An*. *stephensi* in Ethiopia to *Wolbachia* detected in long-established *An. stephensi* populations. As an endosymbiont of invasive mosquitoes, the *Wolbachia* population should mirror a lower level of diversity in its invasive host population relative to the corresponding host source population [43, 44]. A study in pre-print from the state of Tamil Nadu, India, detected four haplotypes of *Wolbachia 16S*, and in eastern Ethiopia we detected three haplotypes. While the SM006 sequence was found in supergroup B that also included the India sequences, we did not have enough sequence resolution to provide definitive evidence of a shared recent ancestor between *An. stephensi Wolbachia* from Ethiopia and India. Further testing would require additional sampling and congruent multilocus genetic data.

There are limitations to using only a PCR assay for detection. Multiple studies have been published regarding *Wolbachia* infection and the detection of *16S*, *wsp*, and MLST. Jeffries et al. [20] discussed how extracting RNA from the mosquitoes would increase the chance of detecting actively expressed *Wolbachia* genes. This could increase the effectiveness of the PCR assays, as they would amplify actively expressed genes rather than the scenarios below. A recent systematic review discussed the different scenarios in which *Wolbachia* could be detected in a mosquito [23]. The authors pointed out that under scenarios such as a mosquito with an endogenized gene from *Wolbachia*, environmental contamination, or a mosquito infected with a nematode infected with *Wolbachia*, *Wolbachia* would be detectable by PCR assay, nested PCR assay, qPCR, and possibly MLST. Additional scenarios were discussed in Chrostek and Gerth [22], where *Wolbachia* sequences could be detected in the gut as a result of the consumption of *Wolbachia*-infected food. In this study, we are able to rule out the scenario in which the mosquitoes were infected with a nematode that was infected with *Wolbachia*, as filarial worms are infected with *Wolbachia* in supergroups C and D. Environmental contamination is a possibility, as *Aedes spp.* share similar breeding habits with *An. stephensi* in eastern Ethiopia. However, other mosquitoes have not been tested for *Wolbachia* in eastern Ethiopia, so we cannot rule this out. For sample SM006, the integration of a *Wolbachia* gene into the host genome is unlikely, as it is not likely that multiple genes would integrate into the host genome [23]. In the scenario that a mosquito is infected with *Wolbachia*, methods such as FISH, transmission electron microscopy (TEM), and Giemsa staining (GIEMSA) must be used to detect the active *Wolbachia* in the ovaries [23]. This suggests that future studies aiming to detect *Wolbachia* in mosquitoes should aim to perform one of these visual methods in addition to molecular assays.

If the detection of *Wolbachia 16S* and *coxA* represents true infection, then our findings provide helpful information about the strains present in *An. stephensi* in eastern Ethiopia. These are helpful data that can inform the approach to malaria control, should *Wolbachia*-based strategies in *Anopheles* become a reality [13]. Insecticide resistance has been identified in *An. stephensi* to pyrethroids, carbamates, and organophosphates, suggesting the need for alternative methods of vector control to prevent the prevalence of malaria from increasing [8, 9]. Identifying the presence of *Wolbachia 16S* and *coxA* DNA in this invasive vector in eastern Ethiopia warrants further studies, including methods such as reverse transcription qPCR and FISH.

## Conclusions

In this study, we present the first evidence suggesting the presence of a *Wolbachia* population in *An. stephensi* in eastern Ethiopia. The detection of *Wolbachia 16S* and *coxA* DNA in *An. stephensi* in Ethiopia supports the need for further investigation of natural *Wolbachia* infections in this species. This could help inform the development of novel approaches for preventing the spread of malaria resulting from this invasive species in the HOA.

## Supplementary Information

**Additional file 1: Table S1.** Number of wild-caught adults and wild-caught larvae that were reared in an insectary at each collection site. **Table S2.** All primers used in PCR amplification. **Table S3.** Accession numbers used in phylogenetic analysis with mosquito species and the publication referenced. **Fig. S1.** Phylogenetic tree of *Wolbachia 16S* in *Anopheles* species in sub-Saharan Africa, eastern Ethiopia, and India. There were two major clades separated by significant bootstrap values 84 and 78. No other differentiation can be detected in this analysis. *Rickettsia japonica* was used as the outgroup (NR_074459). Ethiopian samples are designated by stars, sequences from sub-Saharan Africa are designated by circles, and sequences from India are designated by triangles.

## Data Availability

All data generated or analyzed during this study are included in this published article and its supplementary information files. Sequences have been deposited in the NCBI GenBank database. The accession numbers for *Wolbachia 16S* are OM751324-OM751326, for *Wolbachia coxA* OM811269, and for *An. stephensi COI* OM801690-OM801708.

## Abbreviations

HOA: Horn of Africa
MLST: Multilocus strain typing
CI: Cytoplasmic incompatibility
*wsp*: *Wolbachia* surface protein
FISH: Fluorescence in situ hybridization
PSC: Pyrethrum spray catch
CDC: Centers for Disease Control and Prevention
PCR: Polymerase chain reaction
ITS2: Internal transcribed spacer 2
NCBI: National Center for Biotechnology Information
BLAST: Basic Local Alignment Search Tool
qPCR: Quantitative PCR
TEM: Transmission electron microscopy
GIEMSA: Giemsa stain

## References

[1] World Malaria Report (2020). 20 years of global progress and challenges.

[2] Faulde MK, Rueda LM, Khaireh BA (2014). First record of the Asian malaria vector *Anopheles stephensi* and its possible role in the resurgence of malaria in Djibouti. Horn of Africa Acta Trop.

[3] Carter TE, Yared S, Gebresilassie A, Bonnell V, Damodaran L, Lopez K (2018). First detection of *Anopheles stephensi* Liston, 1901 (Diptera: Culicidae) in Ethiopia using molecular and morphological approaches. Acta Trop.

[4] Malaria Threats Map. https://apps.who.int/malaria/maps/threats/ (2021).

[5] Balkew M, Mumba P, Dengela D, Yohannes G, Getachew D, Yared S (2020). Geographical distribution of *Anopheles stephensi* in eastern Ethiopia. Parasit Vectors.

[6] Tadesse FG, Ashine T, Teka H, Esayas E, Messenger LA, Chali W (2021). *Anopheles stephensi* mosquitoes as vectors of *Plasmodium vivax* and *falciparum*, Horn of Africa, 2019. Emerg Infect Dis.

[7] Hamlet A, Dengela D, Tongren JE, Tadesse FG, Bousema T, Sinka M, Seyoum A, Irish SR, Armistead JS, Churcher T (2021). Preprint: The potential impact of *Anopheles stephensi* establishment on the transmission of *Plasmodium falciparum* in Ethiopia and prospective control measures. MedRxiv..

[8] Yared S, Gebressielasie A, Damodaran L, Bonnell V, Lopez K, Janies D (2020). Insecticide resistance in *Anopheles stephensi* in Somali Region, eastern Ethiopia. Malar J.

[9] Balkew M, Mumba P, Yohannes G, Abiy E, Getachew D, Yared S (2021). Correction to: An update on the distribution, bionomics, and insecticide susceptibility of *Anopheles stephensi* in Ethiopia, 2018–2020. Malar J.

[10] Baldo L, Dunning Hotopp JC, Jolley KA, Bordenstein SR, Biber SA, Choudhury RR (2006). Multilocus sequence typing system for the endosymbiont *Wolbachia pipientis*. Appl Environ Microbiol.

[11] Crawford JE, Clarke DW, Criswell V, Desnoyer M, Cornel D, Deegan B (2020). Author correction: efficient production of male *Wolbachia*-infected *Aedes aegypti* mosquitoes enables large-scale suppression of wild populations. Nat Biotechnol.

[12] O'Neill SL, Ryan PA, Turley AP, Wilson G, Retzki K, Iturbe-Ormaetxe I (2018). Scaled deployment of *Wolbachia* to protect the community from dengue and other *Aedes* transmitted arboviruses. Gates Open Res.

[13] Flores HA, O'Neill SL (2018). Controlling vector-borne diseases by releasing modified mosquitoes. Nat Rev Microbiol.

[14] LePage D, Bordenstein SR (2013). *Wolbachia*: can we save lives with a great pandemic?. Trends Parasitol.

[15] Kambris Z, Blagborough AM, Pinto SB, Blagrove MS, Godfray HC, Sinden RE (2010). Wolbachia stimulates immune gene expression and inhibits *Plasmodium* development in *Anopheles gambiae*. PLoS Pathog.

[16] Hughes GL, Koga R, Xue P, Fukatsu T, Rasgon JL (2011). *Wolbachia* infections are virulent and inhibit the human malaria parasite *Plasmodium falciparum* in *Anopheles gambiae*. PLoS Pathog.

[17] Shaw WR, Marcenac P, Childs LM, Buckee CO, Baldini F, Sawadogo SP (2016). *Wolbachia* infections in natural *Anopheles* populations affect egg laying and negatively correlate with *Plasmodium* development. Nat Commun.

[18] Gomes FM, Hixson BL, Tyner MDW, Ramirez JL, Canepa GE, Alves ESTL (2017). Effect of naturally occurring *Wolbachia* in *Anopheles gambiae* s.l. mosquitoes from Mali on *Plasmodium falciparum* malaria transmission. Proc Natl Acad Sci U S A.

[19] Baldini F, Segata N, Pompon J, Marcenac P, Shaw WR, Dabire RK (2014). Evidence of natural *Wolbachia* infections in field populations of *Anopheles gambiae*. Nat Commun.

[20] Jeffries CL, Cansado-Utrilla C, Beavogui AH, Stica C, Lama EK, Kristan M (2021). Evidence for natural hybridization and novel *Wolbachia* strain superinfections in the *Anopheles gambiae* complex from Guinea. R Soc Open Sci.

[21] Walker T, Quek S, Jeffries CL, Bandibabone J, Dhokiya V, Bamou R (2021). Stable high-density and maternally inherited *Wolbachia* infections in *Anopheles moucheti* and *Anopheles demeilloni* mosquitoes. Curr Biol.

[22] Chrostek E, Gerth M (2019). Is *Anopheles gambiae* a Natural Host of *Wolbachia*?. MBio.

[23] InaciodaSilva LM, Dezordi FZ, Paiva MHS, Wallau GL (2021). Systematic review of *Wolbachia* symbiont detection in mosquitoes: an entangled topic about methodological power and true symbiosis. Pathogens.

[24] Sankar GS, Sundari TM, Anand AA. Preprint: Low prevalence of natural *Wolbachia* in major malarial vectors *Anopheles culicifacies* s.l., and *Anopheles stephensi*: a first report. bioRxiv. 2021.

[25] Gillies MT, Coetzee M (1987). A supplement to the *Anophelinae* of Africa south of the Sahara. S Afr Inst Med Res..

[26] Coetzee M (2020). Key to the females of Afrotropical *Anopheles* mosquitoes (Diptera: Culicidae). Malar J.

[27] Djadid ND, Gholizadeh S, Aghajari M, Zehi AH, Raeisi A, Zakeri S (2006). Genetic analysis of rDNA-ITS2 and RAPD loci in field populations of the malaria vector, *Anopheles stephensi* (Diptera: Culicidae): implications for the control program in Iran. Acta Trop.

[28] Mishra S, Sharma G, Das MK, Pande V, Singh OP (2021). Intragenomic sequence variations in the second internal transcribed spacer (ITS2) ribosomal DNA of the malaria vector *Anopheles stephensi*. PLoS ONE.

[29] Werren JH, Windsor DM (2000). *Wolbachia* infection frequencies in insects: evidence of a global equilibrium?. Proc Biol Sci.

[30] Zhou W, Rousset F, O'Neil S (1998). Phylogeny and PCR-based classification of *Wolbachia* strains using wsp gene sequences. Proc Biol Sci.

[31] Lo N, Casiraghi M, Salati E, Bazzocchi C, Bandi C (2002). How many *Wolbachia* supergroups exist?. Mol Biol Evol.

[32] Jolley KA, Bray JE, Maiden MCJ (2018). Open-access bacterial population genomics: BIGSdb software, the PubMLST.org website and their applications. Wellcome Open Res..

[33] Ayala D, Akone-Ella O, Rahola N, Kengne P, Ngangue MF, Mezeme F (2019). Natural *Wolbachia* infections are common in the major malaria vectors in Central Africa. Evol Appl.

[34] Niang EHA, Bassene H, Makoundou P, Fenollar F, Weill M, Mediannikov O (2018). First report of natural *Wolbachia* infection in wild *Anopheles funestus* population in Senegal. Malar J.

[35] Baldini F, Rouge J, Kreppel K, Mkandawile G, Mapua SA, Sikulu-Lord M (2018). First report of natural *Wolbachia* infection in the malaria mosquito *Anopheles arabiensis* in Tanzania. Parasit Vectors.

[36] Jeffries CL, Lawrence GG, Golovko G, Kristan M, Orsborne J, Spence K (2018). Novel *Wolbachia* strains in *Anopheles* malaria vectors from sub-Saharan Africa. Wellcome Open Res.

[37] Tongkrajang N, Ruenchit P, Tananchai C, Chareonviriyaphap T, Kulkeaw K (2020). Molecular identification of native *Wolbachia pipientis* in *Anopheles minimus* in a low-malaria transmission area of Umphang Valley along the Thailand-Myanmar border. Parasit Vectors.

[38] Wong ML, Liew JWK, Wong WK, Pramasivan S, Mohamed Hassan N, Wan Sulaiman WY (2020). Natural *Wolbachia* infection in field-collected *Anopheles* and other mosquito species from Malaysia. Parasit Vectors.

[39] Katoh K, Rozewicki J, Yamada KD (2019). MAFFT online service: multiple sequence alignment, interactive sequence choice and visualization. Brief Bioinform.

[40] Wane P. Maddison DRM: Mesquite: a modular system for evolutionary analysis. 2021. http://www.mesquiteproject.org.

[41] Stamatakis A (2006). RAxML-VI-HPC: maximum likelihood-based phylogenetic analyses with thousands of taxa and mixed models. Bioinformatics.

[42] Rambaut A: Figtree. 1.4. 4 edn: Institute of Evolutionary Biology; 2007.

[43] Lee CC, Lin CY, Tseng SP, Matsuura K, Yang CS (2020). Ongoing coevolution of *Wolbachia* and a widespread invasive ant, *Anoplolepis gracilipes*. Microorganisms..

[44] Wolfe TM, Bruzzese DJ, Klasson L, Corretto E, Lecic S, Stauffer C (2021). Comparative genome sequencing reveals insights into the dynamics of *Wolbachia* in native and invasive cherry fruit flies. Mol Ecol.

[45] Carter TE, Yared S, Getachew D, Spear J, Choi SH, Samake JN (2021). Genetic diversity of *Anopheles stephensi* in Ethiopia provides insight into patterns of spread. Parasit Vectors.

