# Supplementary material for: Wolbachia 16S rRNA haplotypes detected in wild Anopheles stephensi in eastern Ethiopia
Source: Parasit Vectors. 2022 May 24;15:178. doi: 10.1186/s13071-022-05293-9 (PMC9128127; doi:10.1186/s13071-022-05293-9)
Supplement: Supplementary file 1 — Additional file 1: Table S1. Number of wild-caught adults and wild-caught larvae that were reared in an insectary at each collection site. Table S2. All primers used in PCR amplification. Table S3. Accession numbers used in phylogenetic analysis with mosquito species and the publication referenced. Fig. S1. Phylogenetic tree of Wolbachia 16S in Anopheles species in sub-Saharan Africa, eastern Ethiopia, and India. There were two major clades separated by significant bootstrap values 84 and 78. No other differentiation can be detected in this analysis. Rickettsia japonica was used as the outgroup (NR_074459). Ethiopian samples are designated by stars, sequences from sub-Saharan Africa are designated by circles, and sequences from India are designated by triangles. [file 13071_2022_5293_MOESM1_ESM.docx]

**Table S1**

| **Site** | **Wild caught adult** | **Wild caught larvae/insectary reared** |
| --- | --- | --- |
| Erer-Gota | 7 | 13 |
| Dire Dawa | 6 | 44 |
| Godey | 2 | 44 |
| Kebridehar | 2 | 20 |
| Semera | 33 | 13 |
| Total | 50 | 134 |

Number of wild caught adults and wild caught larvae that were reared in an insectary at each collection site.

**Table S2**

| **Primer name** | ***S*pecies** | **Locus** | **Reference** |
| --- | --- | --- | --- |
| 5.8SB | AATCACTCGGCTCGTGGATCG | ITS2 | Djadid et al. 2006 |
| 28SB | ATGCTTAAATTTAGGGGGTAGTC | ITS2 | Djadid et al. 2006 |
| W-Specf | CATACCTATTCGAAGGGATAG | 16S | Werren and Windsor, 2000 |
| W-Specr | AGCTTCGAGTGAAACCAATTC | 16S | Werren and Windsor, 2000 |
| 16SNF | GAAGGGATAGGGTCGGTTCG | 16S | Shaw et al. 2016 |
| 16SNR | CAATTCCCATGGCGTGACG | 16S | Shaw et.al 2016 |
| Wsp-81F | TGG TCC AAT AAG TGA TGA AGA AAC | wsp | Zhou, et.al 1998 |
| Wsp-691R | AAA AAT TAA ACG CTA CTC CA | wsp | Zhou, et.al 1998 |
| gatB_F1 | GAKTTAAAYCGYGCAGGBGTT | gatB | Baldo, et al. 2006 |
| gatB_R1 | TGGYAAYTCRGGYAAAGATGA | gatB | Baldo, et al. 2006 |
| coxA_F1 | TTGGRGCRATYAACTTTATAG | coxA | Baldo, et al. 2006 |
| coxA_R1 | CTAAAGACTTTKACRCCAGT | coxA | Baldo, et al. 2006 |
| hcpA_F1 | GAAATARCAGTTGCTGCAAA | hcpA | Baldo, et al. 2006 |
| hcpA_R1 | GAAAGTYRAGCAAGYTCTG | hcpA | Baldo, et al. 2006 |
| ftsZuniF | GGYAARGGTGCRGCAGAAGA | ftsZ | Lo, et al. 2002 |
| ftsZuniR | ATCRATRCCAGTTGCAAG | ftsZ | Lo, et al. 2002 |
| fbpA_F1 | GCTGCTCCRCTTGGYWTGAT | fbpA | Baldo, et al. 2006 |
| fbpA_R1 | CCRCCAGARAAAAYYACTATTC | fbpA | Baldo, et al. 2006 |
| gatB_F3 | ATTCAYYTAGARCAAGATGCAGG | gatB | Jolley, et al. 2018 |
| gatB_R3 | AAGAGCTCKGAYAAAGCATYBGC | gatB | Jolley, et al. 2018 |
| coxA_F3 | ATGATTGGCKCACCHGAYATGGC | coxA | Jolley, et al. 2018 |
| coxA_R3 | ACTTTTACACCAGTWATMACRCC | coxA | Jolley, et al. 2018 |
| hcpA_F3 | ATTAGAGAAATARCAGTTGCTGC | hcpA | Jolley, et al. 2018 |
| hcpA_R3 | CATGAAAGACGAGCAARYTCTGG | hcpA | Jolley, et al. 2018 |
| fbpA_F3 | GTTAACCCTGATGCYYAYGAYCC | fbpA | Jolley, et al. 2018 |
| fbpA_R3 | TCTACTTCCTTYGAYTCDCCRCC | fbpA | Jolley, et al. 2018 |
| ftsZ_NF-2 | ATGGGCGGTGGTACTGGAAC | ftsZ | Ayala, et al. 2019 |
| ftsZ_NR-2 | AGCACTAATTGCCCTATCTTCT | ftsZ | Ayala, et al. 2019 |
| coxA_NF-2 | TTTAACATGCGCGCAAAAGG | coxA | Ayala, et al. 2019 |
| coxA_NR-2 | TAAGCCCAACAGTGAACATATG | coxA | Ayala, et al. 2019 |

All primers used in PCR amplification.

**Table S3**

| **Accession number** | ***S*pecies** | **Reference** |
| --- | --- | --- |
| MK755775, MK755774, MK755509 | *An. funestus* | Ayala et al. 2019 |
| MK755788 | *An. implexus* | Ayala et al. 2019 |
| MK755787 | *An. hancocki* | Ayala et al. 2019 |
| MK755789 | *An. jebudensis* | Ayala et al. 2019 |
| MK755834 | *An. paludis* | Ayala et al. 2019 |
| MK755786 | *An. gambiae* | Ayala et al. 2019 |
| MK755790 | *An. nigeriensis* | Ayala et al. 2019 |
| MK755797, MK755816 | *An. moucheti* | Ayala et al. 2019 |
| MK755771, MK755770 | *An. coluzzii* | Ayala et al. 2019 |
| MK755773, MK755772 | *An. coustani* | Ayala et al. 2019 |
| MK755829, MK755823, MK755548, MK755546 | *An. nili* | Ayala et al. 2019 |
| MK755791, MK755544 | *An. marshallii* | Ayala et al. 2019 |
| MK755835, MK755556 | *An. vinckei* | Ayala et al. 2019 |
| MK755768, MK755769 | *An. carnevalei* | Ayala et al. 2019 |
| Sample #: D87987, D87075, D87012 | *An. funestus* | Niang et al. 2018 |
| MH596695, MH596703 | *An. arabiensis* | Baldini et al. 2018 |
| MH605279 | *An. coluzzii* | Jeffries et al. 2018 |
| MH605294 | *An. gambiae* | Jeffries et al. 2018 |
| KJ28739, KJ728742, KJ728750, KJ728755, KJ728746, KJ287749, KJ728745 | *An. gambiae* | Baldini et al. 2014 |
| MF944114, MF946612 | *An. gambiae* | Gomes et al. 2017 |
| MW179590 | *An. gambiae* | Jeffries et al. 2020 |
| MT449018, MT449019 | *An. minimus* | Tongkrajang et al. 2020 |
| MN887548, MN887545, MN887547, MN887538, MN887553, MN887552, MN887541 | *An. introlatus* | Wong et al. 2020 |
| MN887566, MN887569, MN887572, MN887567 | *An. hyrcanus* | Wong et al. 2020 |
| MN887559 | *An. macarthuri* | Wong et al. 2020 |
| MN887576 | *An. balabacensis* | Wong et al. 2020 |
| MN887576, MN887573 | *An. barbirostris* | Wong et al. 2020 |
| MN887560, MN887561 | *An. sinensis* | Wong et al. 2020 |
| MN887558 | *An. latens* | Wong et al. 2020 |
| MN268743, MN268744, MN268746 and MN268750 | *An. stephensi* | Preprint: Gowri Sankar et al. 2021 |
| KT382822 | *An. maculatus* | Direct Submission |
| KU255267 | *Dirofilaria immitis* | Direct Submission |
| NR_074459 | *Rickettsia japonica* | Direct Submission |

Accession numbers used in phylogenetic analysis with mosquito species and the publication referenced.


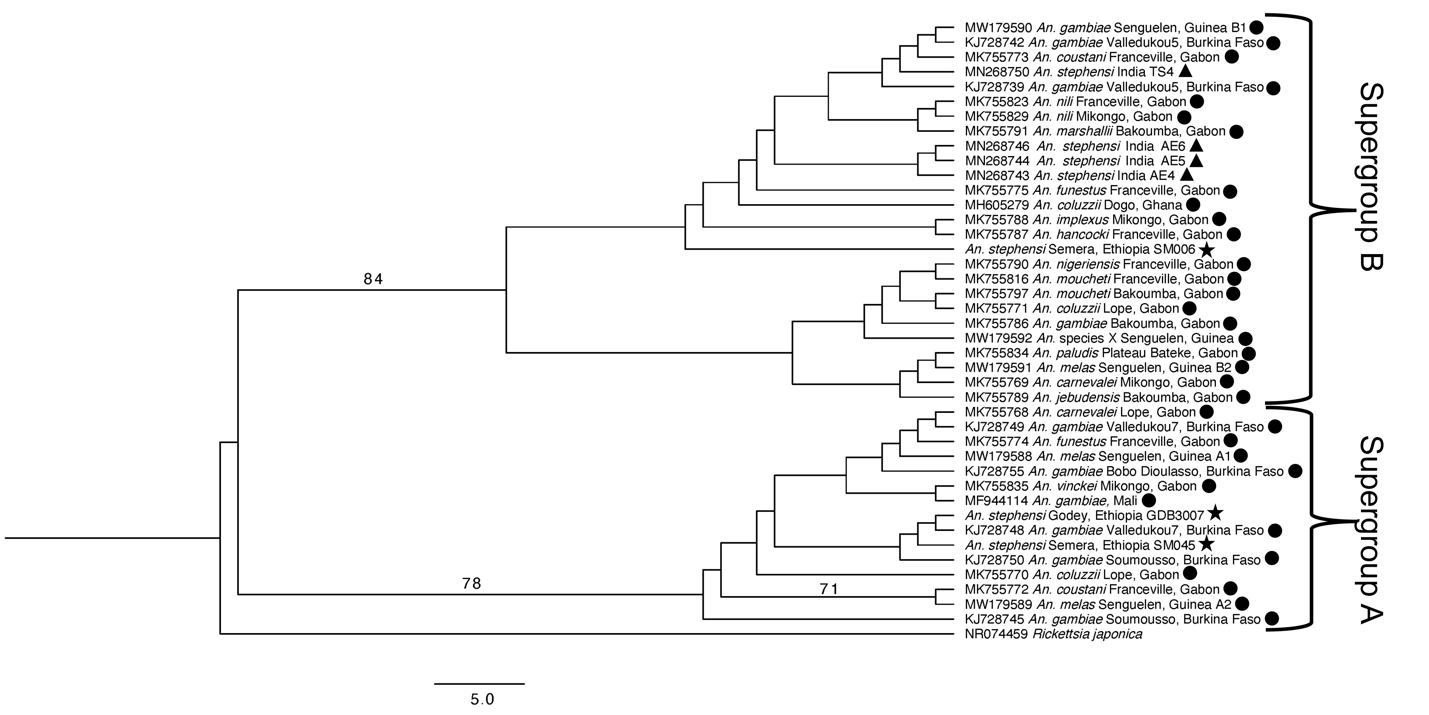


**Fig. S1** Phylogenetic tree of *Wolbachia 16S* in *Anopheles* species in Sub-Saharan Africa, eastern Ethiopia, and India. There were two major clades separated by significant bootstrap values 84 and 78. No other differentiation can be detected in this analysis. *Rickettsia japonica* was used as the outgroup (NR_074459). Ethiopian samples are designated by stars, sequences from Sub-Saharan Africa are designated by circles, and sequences from India are designated by triangles.
